# Validation and assessment of the arabic psychological first aid scale among physicians, nurses, and counselors in Jordan

**DOI:** 10.1186/s40359-025-02428-2

**Published:** 2025-02-05

**Authors:** Basma Eid Alshareef, Othman A. Alfuqaha, Ibraheem B. Maali, Khaled Amer

**Affiliations:** 1 Department of Clinical Psychology, Faculty of Arts and Sciences, AL-Ahliyya Amman University, Amman, 19328 Jordan; 2 Counseling and Mental Health Department, Faculty of Educational Sciences, The World Islamic Sciences & Education University, Amman, 11947 Jordan; 3 Department of General Surgery, School of Medicine, The University of Jordan, Amman, 11942 Jordan

**Keywords:** Healthcare providers, Psychological first aid, Reliability, Validity

## Abstract

**Background:**

Psychological first aid (PFA) involves strategies to tackle problems that occur as a result of disasters. This study aimed to validate the Arabic version of PFA scale through validity (face, content, convergent, discriminant validity, and confirmatory factor analysis) and reliability (Cronbach’s alpha). Furthermore, it aimed to assess the perceived levels of knowledge, skills, and attitudes of PFA among healthcare providers (HCPs). Moreover, it determined the differences between HCPs based on their knowledge, skills, and attitudes of PFA.

**Methods:**

A sample size of 389 HCPs (physicians = 68, nurses = 173, and counselors = 148) was conveniently selected to participate during the period of 10 December 2023 to 10 February 2024 after completing the translated PFA scale into the Arabic language. Descriptive, translation, validation process, and reliability were conducted.

**Results:**

The translation process was satisfied. The content validity index was 0.91, yielding proper clarity of items. Three constructs were loaded with a total variation of 63.43%, indicating a proper model fit. The goodness-of-fit indices for the PFA model revealed that relative chi-square was 1.47, comparative fit index was 0.91, Tucker-Lewis index was 0.89, and the root mean square of error approximation was 0.046. Cronbach alpha values for knowledge, skills, and attitudes of PFA were 0.90, 0.89, 0.87, respectively. We found that counselors have more knowledge (64.4%), skills (73.4%), and attitudes (73.4%) compared to physicians and nurses. There are significant differences (*p* < 0.001) between the selected HCP groups, particularly for the counselors’ group in terms of knowledge, skills, and attitudes of PFA.

**Conclusions:**

The Arabic PFA scale is valid and reliable among HCPs. The results highlight the emergent need to provide knowledge, skills, and attitudes of PFA interventions among medical professionals, particularly for physicians and nurses. The Arabic version of PFA helps all HCPs in Arab countries to assess, apply, and implement PFA interventions.

**Supplementary Information:**

The online version contains supplementary material available at 10.1186/s40359-025-02428-2.

## Background

In the past few years with COVID-19, earthquakes, and wars, we have realized the need to address psychological issues that individuals, families, and communities experience due to trauma. These have led to more physical and psychological problems including disabilities, psychological and behavioral reactions [1, 2]. We need to be aware of the basic principles of psychological first aid (PFA) to meet the needs of those affected [3]. Thus, we need PFA strategies to prevent long-term psychological distress.

PFA can be defined as a practical approach that involves strategies and interventions to help and support individuals after traumatic events [4]. It aims to reduce distress and provide safety to trauma patients through the application of specific psychological interventions and strategies [5]. So, traumatized people should seek help based on PFA strategies. These strategies are built on three core dimensions: knowledge, skills, and attitudes. They are identified as protective factors against traumatic events and crises [6]. Providers of PFA interventions should understand and apply these three core dimensions well. Knowledge includes concepts and information about crises, stress, post-traumatic stress disorder (PTSD), depression, and principles of suicidality. Skills include self-efficacy, active listening, prioritize needs, teach breathing techniques, and interpret meanings and feelings. Attitudes integrate beliefs and motivations [7, 8]. On the other hand, several barriers prevent traumatized individuals from seeking PFA strategies from healthcare providers (HCPs) including social stigma, fear of treatment, and religious fatalism [9].

Physicians, nurses, and counselors are the first responders to crises and traumatic events [10]. They should implement PFA strategies in their workplace, but a lack of knowledge, skills, and attitudes toward PFA among these professionals can worsen the situation for patients. In Palestine, they did a study and found that nurses who underwent PFA training were more psychologically prepared [11]. In South Korea, they did a study to show the effectiveness of PFA training for school counselors and found it increased their knowledge, skills, and attitudes [12]. In 2023, they did a letter for managing acute stress among physicians through PFA strategies [13]. No studies in the Arabic context are found in this field.

Several models have been developed to implement PFA strategies for traumatic patients [14, 15]. However, limited quantitative scales are noticed to measure the knowledge, skills, and attitudes of PFA. Only one study published by Johns Hopkins PFA training hospital has developed such a scale [8]. This scale has 21 items and is divided into three dimensions: knowledge 9-items, skills 7-items, and attitudes 5-items. It was found to be a reliable and valid tool among police officers [16] and disaster people [17]. This scale has not been translated into the Arabic language.

As mentioned in the literature, there is no PFA scale in the Arabic context to measure knowledge, skills, and attitudes. Therefore, translating and validating the PFA scale into Arabic language is necessary for Arabic researchers. This will enable them to develop and implement models and strategies based on this study and provide insights for HCPs to assess their knowledge, skills, and attitudes of PFA among three important groups which are physicians, nurses, and counselors. Moreover, the differences between the selected HCP groups in terms of knowledge, skills, and attitudes of PFA were also studied to determine which group needs more attention from researchers and policymakers to set interventions, psychological programs and strategies to be applied to them first.

## Methods

### Aims

This study aimed to validate the Arabic version of PFA through validity (face, content, convergent, and discriminant validity, confirmatory factor analysis (CFA), and reliability (Cronbach’s alpha). Furthermore, it aimed to assess the perceived levels of knowledge, skills, and attitudes of PFA among HCPs. Moreover, it determined the differences between them based on their knowledge, skills, and attitudes of PFA.

### Study design and characteristics of participants

We performed a cross-sectional methodological design. We selected three medical professionals (physicians, nurses, and counselors) involved in providing PFA strategies and having direct contact with traumatic patients in hospitals and refugee organizations. The inclusion criteria were medical professionals limited to physicians, nurses, and counselors; have different levels of experience and educational levels; work at different sites at hospitals; and willing to participate. The exclusion criteria were not the selected HCP groups, those unwilling to participate, and those with expertise in PFA such as those who were working in psychiatric and traumatic units.

### Settings

The study population included physicians, nurses, and counselors, and the sample size was determined using G*Power software and guided by previous literature [18], which suggested a minimum of 100 participants from nurses, 100 participants from counselors, and 60 participants from physicians. Studies have also suggested including 5–10 participants per item, based on EFA and CFA recommendations. We conveniently distributed a total of 200 paper-based surveys among nurses and counselors and 100 paper-based surveys among physicians during the period from 10 December 2023 to 10 February 2024. The response rates from nurses and counselors were 86.5% and 74%, respectively, while from physicians, it was 68%. During the distribution process, researchers visited Jordan University Hospital conveniently to collect data from physicians and nurses, while counselors were contacted via regular meetings at a university. We explained the aim of the study, provided the consent form, and highlighted that there was no obligation to complete the questionnaire. On average, participants took approximately 3 min to complete the survey. The Arabic version of the PFA scale was administered using a 5-point Likert-type scale, from “Strongly Disagree” to “Strongly Agree”. The higher score indicates the higher knowledge, skills, and attitudes of PFA according to the Johns Hopkins PFA training guidelines.

### Translation and validation process

The Johns Hopkins PFA training hospital developed a scale to assess knowledge, skills, and attitudes of PFA [8]. This scale involves 21 items, divided into three constructs: knowledge (9 items), skills (7 items), and attitudes (5 items). The translation process started with the translation of the English version into Arabic language by researchers. Then, the Arabic version of PFA scale was examined by specialized experts in Arabic-English translation to ensure its appropriateness. Following this, the Arabic version was retranslated back into the English language through other dependent experts in English Arabic to verify its consistency with the original scale [19]. Appendix A.

After completing the translation process, we administered the translated Arabic version of the PFA scale to 25 participants who were not part of the study sample. This was done to evaluate their comprehension of the items and assess the clarity of each item, a process known as face validity. On the other hand, content validity was evaluated by several experts specialized in psychology (two experts), medicine (two experts), and counseling (two experts). Utilizing a three-point Likert-type scale (essential, not essential, I don’t know), we requested them to determine the content validity ratio (CVR). Content validity index (CVI) scored based on relevance and clarity of items, we used a four-point Likert scale. Furthermore, construct validity was examined through explanatory factor analysis (EFA) methods of intercorrelation varimax rotation (coefficients > 0.30), eigenvalues (> 1), Kaiser-Meyer-Olkin (KMO) measure (> 0.70), and Bartlett’s test of sphericity (*p* < 0.05). Convergent and discriminant validities were assessed by average variance extracted (AVE) and squared root of the AVE, respectively. Moreover, we performed CFA by AMOS program v.26 to illustrate the relationships between items with their constructs and to assess model fit indices. Finally, Cronbach’s alpha values were calculated separately for the three constructs of PFA scale to measure the internal consistency.

### Statistical analysis

After entering the data into SPSS v.23, we checked outliers, normality, and missing data. In the validation process, we relied on the CVR results according to Lawshe’s Table, which indicates that 0.83 from 6 experts represents an excellent level of agreement [20]. The validity process was assessed using global guidelines including intercorrelation varimax rotation (with coefficients > 0.30), eigenvalues (> 1), KMO (> 0.70), Bartlett’s test of sphericity (*p* < 0.05), AVE (> 0.50) [21], relative Chi-square (χ2/df ratio) (< 3), root mean square of error approximation (RMSEA) (< 0.05), comparative fit index (CFI ≥ 0.90), and Tucker-Lewis index (TLI) (> 0.85) [22]. Cronbach’s alpha values greater than 0.70 indicate internal consistency [23]. Then, we performed a descriptive analysis to calculate the perceived levels of knowledge, skills, and attitudes of PFA among the three HCP groups (physicians, nurses, and counselors) separately. After that, we used the ANOVA test and Sheffe multiple comparisons to determine the differences between the selected HCP groups. Finally, the Eta square value (0.01 small effect, 0.06 medium effect, and ≥ 0.14 large effect size) was used to assess the effect size [24].

### Ethics approval and consent to participate

#### Ethical approval

was obtained from both Jordan University Hospital (Approval No: https://doi.org/10/2023/30560) and the World Islamic Sciences and Education University (approval No: 5/1/9/925). We prepared an envelope containing informed consent, demographic information, and the Arabic version of the PFA scale. Before the distribution process, written informed consent to participate was obtained from all participants in the study by filling in their names and live signatures.

## Results

### Demographic information

The total number of physicians who participated in this study was 68, with more than half of them being male, single, and holding a bachelor’s degree. Among nurses, females who were married and held a bachelor’s degree, with over 10 years of experience, were the most predominant. Among counselors, females who were single, held a bachelor’s degree, and had between 0 and 5 years of experience were common. Surprisingly, more than two-thirds of all participants (physicians, nurses, and counselors) did not receive PFA training courses (Table 1).

**Table 1:** Demographic data for nurses, physicians, and counselors

Demographic information	Descriptive	Nurses (*N* = 173) *n* (%)	Physicians (*N* = 68) *n* (%)	Counselors (*N* = 148) *n* (%)
Gender	Male Female	70 (40.5) 103 (59.5)	43 (63.2) 25 (36.8)	16 (10.8) 132 (89.2)
Marital status	Single Married Other	41 (23.7) 123 (71.1) 9 (5.2)	40 (58.8) 27 (39.7) 1 (1.5)	125 (84.5) 17 (11.5) 6 (4.1)
Age (years)	M ± SD	36.32 ± 7.8	26.62 ± 3.58	22.39 ± 3.49
Educational level	Bachelor Master PhD	160 (92.5) 8 (4.6) 5 (2.9)	38 (55.9) 29 (42.6) 1 (1.5)	146 (98.6) 2 (1.4) 0 (0)
Experience level (Years)	0–5 6–10 > 10	26 (15) 47 (27.2) 100 (57.8)	34 (50) 26 (38.2) 8 (11.8)	107 (72.3) 34 (23) 7 (4.7)
Did you receive training regarding PFA	Yes No	49 (28.3) 124 (71.7)	13 (19.1) 55 (80.9)	35 (23.6) 113 (76.4)

**Notes**: n (%): Number (Percentage), M ± SD: Mean ± Standard deviation

### Translation and validation process

Forward and backward translations were conducted with experts in English-Arabic translation. All experts reached an agreement on the final Arabic version of the PFA scale. All 25 participants (pilot study), upon completing the Arabic version of the PFA, indicated that all items were clear, easy to read, and none were perceived as vague. Based on 6 experts specialized in psychology, medicine, and counseling, agreed with a percentage of 0.89 on the essentiality of the Arabic PFA items, reaching a CVR above the cutoff score according to Lawshe’s Table (0.83). The CVI revealed a percentage of (0.91) based on the clarity and relevance of items. Some experts suggested amendments to certain words to make them more suitable, but none of them suggested erasing items.

The intercorrelation varimax rotation revealed scores above 0.30 for all items. The KMO result was 0.94. Bartlett’s test of sphericity yielded (*x* = 5411.81, df = 210, *p* < 0.001). The cumulative loading on three constructs with eigenvalues above 1 accounted for 63.43% of the total variance (Table 2). These findings suggest the construct validity of the Arabic version of PFA scale.

**Table 2:** Intercorrelation varimax rotation of the translated arabic version of PFA scale

Items	Factor 1 Knowledge	Factor 2 Skills	Factor 3 Attitudes
Knowledge item # 1	**0.64**	0.02	0.03
Knowledge item # 2	**0.67**	0.18	0.11
Knowledge item # 3	**0.73**	0.21	0.21
Knowledge item # 4	**0.67**	0.09	0.04
Knowledge item # 5	**0.74**	0.01	0.07
Knowledge item # 6	**0.67**	0.22	0.02
Knowledge item # 7	**0.69**	0.04	0.21
Knowledge item # 8	**0.70**	0.21	0.11
Knowledge item # 9	**0.67**	0.06	0.09
Cronbach Alpha	**0.90**		
Average variance extracted (AVE)	**0.74**		
Squared root of AVE	**0.86**		
Skills item # 1	0.16	**0.70**	0.27
Skills item # 2	0.17	**0.70**	0.18
Skills item # 3	0.07	**0.68**	0.11
Skills item # 4	0.19	**0.70**	0.15
Skills item # 5	0.06	**0.69**	0.20
Skills item # 6	0.11	**0.78**	0.19
Skills item # 7	0.14	**0.74**	0.24
Cronbach Alpha		**0.89**	
Average variance extracted		**0.73**	
Squared root of AVE		**0.86**	
Attitude item # 1	0.06	0.06	**0.71**
Attitude item # 2	0.19	0.27	**0.73**
Attitude item # 3	0.22	0.25	**0.78**
Attitude item # 4	0.09	0.19	**0.77**
Attitude item # 5	0.29	0.32	**0.63**
Cronbach Alpha			**0.87**
Average variance extracted			**0.76**
Squared root of AVE			**0.87**
Initial eigenvalues	**10.46**	**1.68**	**1.17**
Percentages of variance explained	**49.82**	**8.03**	**5.58**
Cumulative variance	**49.82**	**57.85**	**63.43**

All values of AVE were above 0.50 which indicates convergent validity of the Arabic version of PFA scales. The values of the squared root of AVE were higher than the values among intercorrelations constructs in CFA, indicating discriminant validity.

### Confirmatory factor analysis

**Fig. 1:**
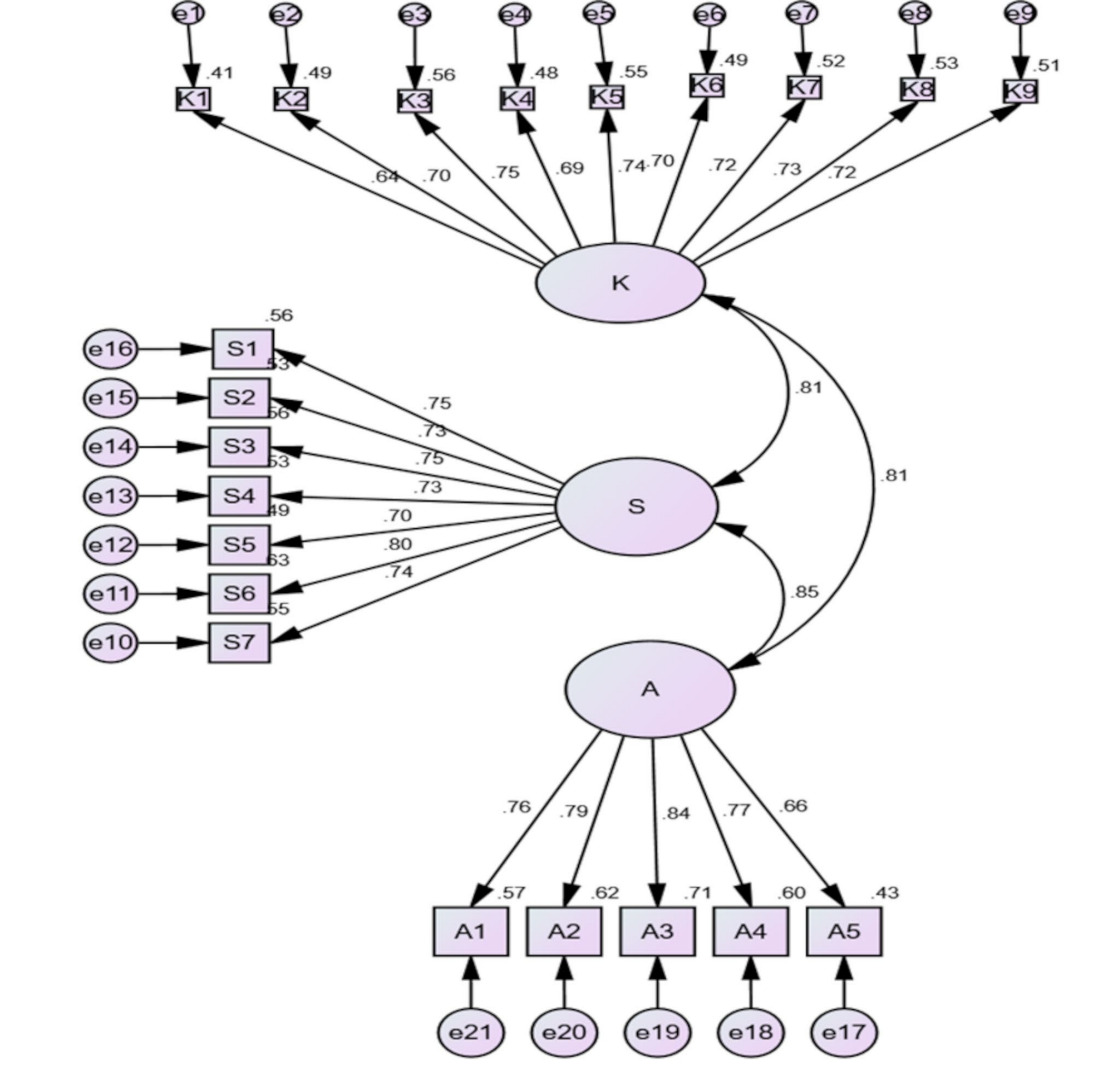
CFA model for the Arabic PFA scale. K: knowledge, S: skills, A: attitude

We entered 21 items into the AMOS program (V.26) to assess factor loadings between the constructs of the PFA scale (knowledge, skills, attitude) and their items. The factor loadings for knowledge construct items ranged between 0.64 and 0.75, for skills construct items ranged between 0.73 and 0.80, and for attitude construct items ranged between 0.66 and 0.84. These intercorrelation values indicated an excellent relationship (Fig. 1). The goodness-of-fit indices for the PFA model revealed that relative chi-square was 1.47, CFI was 0.91, TLI was 0.89, and RMSEA was 0.046. These numbers indicated that the PFA scale is valid by CFA.

### Internal consistency

The reliability results in Table 2 indicate that knowledge, skills, and attitudes were 0.90, 0.89, and 0.87, respectively, supporting the reliability of PFA constructs.

### Perceived levels of knowledge, skills, and attitudes of PFA and the differences between HCP groups

Counselors exhibited the highest mean scores based on knowledge (3.22 ± 0.65), skills (3.67 ± 0.70), and attitudes (3.67 ± 0.78) of PFA compared to nurses and physicians. Surprisingly, physicians had the lowest mean score on knowledge (2.84 ± 0.63) and skills (3.14 ± 0.63) of PFA. The attitude of PFA was the lowest among nurses (3.24 ± 0.79) compared to other HCP groups. Percentages confirmed that counselors have higher knowledge (64.4%), skills (73.4%) and attitudes (73.4%) compared to other groups. Physicians have less percentage of knowledge (56.8%) and skills (62.8%). While nurses have a lower percentage of attitudes (64.8%) regarding PFA (Table 3).

**Table 3:** Means sores for PFA constructs and the differences between HCP groups

PFA		N	M	Percentage	SD	f-distribution	p-value	eta square
Knowledge	Nurses	173	2.88	57.6%	0.75	11.51	0.001***	0.056
	Physicians	68	2.84	56.8%	0.63			
	Counselor	148	3.22	64.4%	0.65			
Skills	Nurses	173	3.19	63.8%	0.77	21.74	0.001***	0.101
	Physicians	68	3.14	62.8%	0.63			
	Counselor	148	3.67	73.4%	0.70			
Attitudes	Nurses	173	3.24	64.8%	0.79	13.24	0.001***	0.064
	Physicians	68	3.32	66.4%	0.61			
	Counselor	148	3.67	73.4%	0.78			

**Notes.** M: Mean, SD: Standard deviation, ***Significance level < 0.001

There were statistically significant differences (*p* < 0.001) in the levels of knowledge, skills, and attitudes of PFA among the three selected HCP groups (Table 3). Sheffe’s multiple comparison results showed that the counselor group is the most associated with knowledge, skills, and attitudes of PFA. However, no significant differences were observed between physicians and nurses based on PFA constructs. Eta square values indicated a medium effect size between PFA constructs and HCP groups.

## Discussion

This study explores the knowledge, skills, and attitudes of PFA among three important HCP groups which are physicians, nurses, and counselors. We found that the Arabic PFA scale is valid and reliable among the selected participants through the translation process, face, content, construct, convergent, and discriminant validity and CFA and through reliability which was assessed by Cronbach’s alpha. Moreover, we found that counselors have more knowledge, skills, and attitudes than physicians and nurses. Finally, there are statistically significant differences between the HCP groups, especially for the counselors group.

This is the first study in Jordan to test the validity and reliability of the PFA scale across three important HCP groups. After the translation process, experts agreed on the final Arabic PFA scale and confirmed its clarity, comprehensibility, and cultural appropriateness for Arabic-speaking populations. Among 101 Chilean populations, a study was conducted to validate a Spanish behavior questionnaire used in PFA interventions. After demonstrating both validity by CFA and reliability by McDonald’s omega, they found 18 items divided into 5 constructs [25]. In the Netherlands, they did a validation study among 270 physical therapists for a Determinants of Implementation Behavior Questionnaire (DIBQ) to measure knowledge, skills, and beliefs related to behavioral change. They demonstrated CFA analysis and found that the tool has 93 items distributed across 18 domains, explaining a total of 63.3% of the variance [26]. Focusing primarily on HCPs caring for trauma patients, determining that a global PFA scale, such as the one provided by Johns Hopkins Hospital, and providing fewer items will effectively utilize PFA interventions.

The face, content, construct, convergent, discriminant, and CFA validity met the recognized criteria levels. The pilot participants confirmed the clarity and comprehensibility of the Arabic PFA scale. Specialized experts confirmed that it measures what it intends to measure. Moreover, CVI, CVR, KMO, intercorrelations, and CFA exceeded the acceptance levels. The total variance was 63.43% divided into three constructs each with an eigenvalue more than 1. Comparing our results with a study conducted in Turkey, in which they developed a self-efficacy PFA scale through factor analysis. They found that only one-factor loading through 35 items with a total variation of 58.47% [27]. Our model was chosen over potential alternatives as it aligned with the theoretical framework of the original scale and demonstrated superior model fit based on cumulative variance, EFA, and CFA results. Furthermore, our translation of the PFA scale into Arabic incorporated cultural adoption, targeted three important HCPs, and adequate sample size.

Surprisingly, more than two-thirds of participants did not receive any PFA training on specific strategies and interventions. This raises questions about the priority of first aid among HCPs who are in contact with traumatic patients. This is consistent with a previous study that found more than 90% of nurses did not receive PFA training [28]. This highlights the need for policymakers to train all HCPs in various areas to be prepared for current crises like COVID-19 and earthquakes [1, 29]. It is widely known that model-based strategies and interventions promote knowledge, skills, and mental health of traumatic patients during such crises [30, 31].

Interestingly, our findings showed that counselors have more knowledge, skills, and attitudes than other HCP groups. This may be related to specific courses they took during their education or because of the nature of their work which involves patients who have lived traumatic experiences. Physicians have lower levels of knowledge and skills despite treating patients with traumatic illnesses such as chronic diseases or cancer. Nurses have a lower percentage of attitudes, so there is an urgent need for specific counseling programs on PFA strategies and interventions, especially for physicians and nurses. Other important factors that let physicians and nurses lag behind counselors are contextual factors such as professional roles, training opportunities, and work environments. Nurses and physicians seem to have less professional status [32], less concern about training in PFA interventions [29], and more job demands-resources in the workplace than counselors [33]. Comparing our results with previous studies, almost one-third of health professionals in Brunei Darussalam had moderate levels of knowledge and attitude which is similar to our findings [34]. In another study, they found a significant increase in knowledge, skills, and attitudes after PFA educational program [35].

We found that counselors have more knowledge, skills, and attitudes than physicians and nurses and this was statistically significant. This can be explained by several factors including the focus of counselors on mental health problems, psychological issues, and cognitive/behavioral problems [36]. Another explanation is related to age groups and types of illness in traumatized individuals. For example, counselors work with different age groups and psychological disorders whereas nurses and physicians work with physical symptoms across different age groups. Counselors also work in different settings such as schools, refugee organizations, and family care institutions whereas other healthcare professionals work in hospitals [37, 38]. This is opposite to previous studies which showed that nurses and university students have more knowledge and skills after training on PFA interventions [28, 39].

This study has several strengths, first validation of the PFA scale into the Arabic language makes it useful for future studies and researchers to focus on the preparation of PFA strategies. Second, the three important selected HCP groups were included to explore their knowledge, skills, and attitudes toward PFA. Moreover, using EFA and CFA analysis on the same dataset would lead to overfitting our findings. However, the use of surveys by cross-sectional methods and the sample size could limit the generalizability of the data, and these should be considered as limitations.

## Conclusion

We found that the Arabic version of the PFA scale is valid and reliable, as it achieved the required levels of translation, face validity, CVI, CVR, KMO, EFA, and CFA. Counselors have higher levels of knowledge, skills, and attitudes compared to physicians and nurses, despite most of them not having any PFA training. Future studies are guaranteed to build strategies and interventions for PFA based on the Arabic PFA scale. We recommend longitudinal studies on PFA training effectiveness to understand how it influences HCPs’ coping mechanisms over time. We also recommend that the Arabic PFA scale be applied to other professionals who have direct contact with trauma patients. Policymakers, hospitals, and educational institutions in Jordan should integrate specific psychological strategies and training courses for physicians, nurses, and counselors based on PFA knowledge, skills, and attitudes. These strategies and courses will improve the mental health of survivors in crisis and patients requiring urgent psychological intervention.

## Electronic supplementary material

Below is the link to the electronic supplementary material.

Supplementary Material 1

## Data Availability

The data that support the findings of this study are not openly available due to reasons of sensitivity and are available from the corresponding author upon reasonable request. Data are located in controlled access data storage at Jordan University Hospital (juhosp@ju.edu.jo, phone #: (00962 (6) 535 3444).

## Abbreviations

PFA: Psychological first aid
HCPs: Healthcare providers
CVR: Content validity ratio
CVI: Content validity index
KMO: Kaiser-Meyer-Olkin
AVE: Average variance extracted
CFA: Confirmatory factor analysis
