# Supplementary material for: Validation and assessment of the arabic psychological first aid scale among physicians, nurses, and counselors in Jordan
Source: BMC Psychol. 2025 Feb 5;13:103. doi: 10.1186/s40359-025-02428-2 (PMC11800430; doi:10.1186/s40359-025-02428-2)
Supplement: Supplementary file 1 — Supplementary Material 1 [file 40359_2025_2428_MOESM1_ESM.docx]

Material

**Knowledge, skills, and attitudes questionnaire of Psychological First Aid (PFA)**

Adapted from Johns Hopkins PFA training evaluation study

Ref: McCabe, O. L., Semon, N. L., Thompson, C. B., Lating, J. M., Everly Jr, G. S., Perry, C. J., ... & Links, J. M. (2014). Building a national model of public mental health preparedness and community resilience: validation of a dual-intervention, systems-based approach. *Disaster medicine and public health preparedness*, *8*(6), 511-526.

A self-report, 5-point Likert-scale composed of 18 items, organized by knowledge (7 items), skills (7 items), and attitudes (3 items) facilitative of stressful events response.

|  | item | Strongly disagree  1 | Disagree  2 | Neutral  3 | Agree  4 | Strongly agree  5 |
| --- | --- | --- | --- | --- | --- | --- |
| Knowledge: self-reported understanding of: | | | | | | |
|  | The concept of stress, crisis |  |  |  |  |  |
|  | The logic of extending PFA training |  |  |  |  |  |
|  | Principles of the stress response & relaxation training |  |  |  |  |  |
|  | Characteristics of acute stress disorder |  |  |  |  |  |
|  | Predictor of PTSD |  |  |  |  |  |
|  | Principles of screening for depression and suicidality, psychosis |  |  |  |  |  |
|  | 5 core components of PFA |  |  |  |  |  |
|  | Important questions to ask before deployment |  |  |  |  |  |
|  | 4 self-care practices for own selves |  |  |  |  |  |
| Skills: perceived self-efficacy, proficiency and ability to: | | | | | | |
|  | Use listening skills to build rapport |  |  |  |  |  |
|  | Discern meanings and feelings from statements |  |  |  |  |  |
|  | Prioritize the needs of a distressed one |  |  |  |  |  |
|  | Differentiate severe from moderate distress |  |  |  |  |  |
|  | Teach/demonstrate diaphragmatic breathing |  |  |  |  |  |
|  | Respond to mental health referral needs |  |  |  |  |  |
|  | Overall PFA self-efficacy |  |  |  |  |  |
| Attitudes: endorsed attitudes, beliefs, and motivations: | | | | | | |
|  | Likelihood of a stress situation |  |  |  |  |  |
|  | Likelihood of stress-caused need for PFA |  |  |  |  |  |
|  | Perceived effectiveness of correctly applied PFA |  |  |  |  |  |
|  | Current adequacy of crisis mental health preparedness |  |  |  |  |  |
|  | Willingness to be a listener and supporter |  |  |  |  |  |
